# Defensive Gin-Trap Closure Response of Tenebrionid Beetle, *Zophobas atratus*, Pupae

**DOI:** 10.1673/031.012.13401

**Published:** 2012-11-11

**Authors:** Toshio Ichikawa, Toshiaki Kurauchi, Yoshifumi Yamawaki

**Affiliations:** ^1^ Department of Biology, Faculty of Sciences, Kyushu University, Fukuoka 812-8581, Japan; ^2^ Basic biology, Graduate School of System Life Sciences, Kyushu University, Fukuoka 812-8581, Japan

**Keywords:** defensive behavior, mechanoreceptor, predators, sensilla

## Abstract

Pupae of the beetle *Zophobas atratus* Fab. (Coleoptera: Tenebrionidae) have jaws called gin traps on the lateral margin of their jointed abdominal segments. When a weak tactile stimulation was applied to the intersegmental region between the two jaws of a gin trap in a resting pupa, the pupa rapidly closed and reopened single or multiple gin traps adjacent to the stimulated trap for 100200 ms. In response to a strong stimulation, a small or large rotation of the abdominal segments occurred after the rapid closure of the traps. Analyses of trajectory patterns of the last abdominal segment during the rotations revealed that the rotational responses were graded and highly variable with respect to the amplitudes of their horizontal and vertical components. The high variability of these rotational responses is in contrast with the low variability (or constancy) of abdominal rotations induced by the tactile stimulation of cephalic and thoracic appendages. Since the closed state of the gin traps lasts only for a fraction of a second, the response may mainly function to deliver a “painful” stimulus to an attacker rather than to cause serious damage.

## Introduction

Holometabolous insect larvae metamorphose into adults through the pupal stage. Although pupae do not move at all or their locomotive capacity is greatly restricted, they are usually protected by physical, chemical, or biological (behavioral) mechanisms (Hinton 1955). The pupae of many Coleoptera and some Lepidoptera species are armed with heavily sclerotized projections or jaws near the intersegmental regions of adjacent abdominal segments (Hinton 1946, 1952). The pupae often swing or rotate their abdomen in response to tactile stimulation of their appendages (Hollis 1963; Askew and Kurtz 1974), while they rapidly close the jaws in response to the stimulation of the intersegmental regions of the abdominal segments (Hinton 1946; Wilson 1971; Eisner and Eisner 1992). Hinton (1946) coined the term “gin trap” to describe the pinching device.

Sensory and neuronal mechanisms of the defensive response have been examined in the pupae of hawkmoths (Bate 1973a, b, c; Levine et al. 1985; Waldrop and Levine 1989, 1992; Lemon and Levine 1997). Tactile stimulation of the mechanosensory hairs located within small pits of the gin traps on the abdomen induces rapid bending of the abdomen toward the side of stimulation and the closing of one or more of the gin traps (Bate 1973b). Since the pupae of many Coleopteran insects (i.e., beetles) have highly developed gin trap structures (Hinton 1946; Bouchard and Steiner 2004), physiological and behavioral studies of these insects may provide insight into the functions and evolution of pupal defensive mechanisms. Robust gin-trap closure responses have been observed in the tenebrionid beetle *Tenebrio molitor* (Hinton 1946; Wilson 1971), although the functional mechanism is largely unknown.

To clarify these issues, a series of morphological, physiological, and behavioral studies of the pupal defensive responses were performed using the pupae of a large tenebrionid beetle *Zophobas atratus* Fab. (Coleoptera: Tenebrionidae) from Central America (Tschinkel 1981). Many campaniform sensilla (strain sensors) were scattered over almost all parts of the pupal cuticle, including appendages and intersegmental membrane. This type of mechanoreceptive sensilla plays a role in triggering the gin-trap closure response as well as the abdominal rotation response (Kurauchi et al. 2011); the latter is induced by stimulating a cephalic or thoracic appendage and is characterized by relatively constant trajectory patterns of abdominal rotations as described in the Ichikawa et al. 2012. In the present study, pupal gin-trap closure responses were found to often accompany abdominal rotations with a variable trajectory pattern.

## Materials and Methods

### Animals

Giant mealworms, Z *atratus*, were purchased as completely grown larvae from a local supplier. The detritivorous or omnivorous larvae were kept under crowded conditions in a mixture of peat moss and sawdust and were fed fresh Japanese radishes. Individual larvae were isolated in a plastic cup for pupation. The pupae were maintained at 26 ± 1° C under a 16: 8 L:D photoperiod.

### High-speed photography

One-day-old pupae were usually used for the analysis of the gin-trap closure responses. The dorsal part of the thorax of a pupa was fixed to an edge of a horizontal plane of a rectangular block with melted paraffin, and the block was placed on a platform so that the horizontal plane faced upward or downward. Captures and analyses of high-speed movies (200 frames/s) were made as described in the Ichikawa et al. 2012.

### Mechanical stimulation

Gin-trap closure responses were usually induced by manually brushing the sensitive area of the intersegmental membrane near the gin trap with a tip of a writing brush in order to prevent the soft intersegmental membrane from being damaged by repetitive mechanical stimulation. Although the force of manual brushing could not be controlled precisely, the force was estimated using a calibrated strain gauge; weak and strong brushings were approximately 0.3 and 1.5 mN, respectively. A thin nylon filament or nichrome wire with a known bending force (Kurauchi et al. 2011) was sometimes used to determine the timing of stimulation and latency of the response. A tibial segment (0.4 mm in diameter) from an adult *Z. atratus* was also used to test whether the closed state of a gin trap was prolonged, when the trap could successfully pinch the tubular segment mimicking an appendage of a potential enemy. To induce an abdominal rotational response, a weak brushing was applied to the distal portion of the middle-leg femur.

## Results

**Figure 1.**
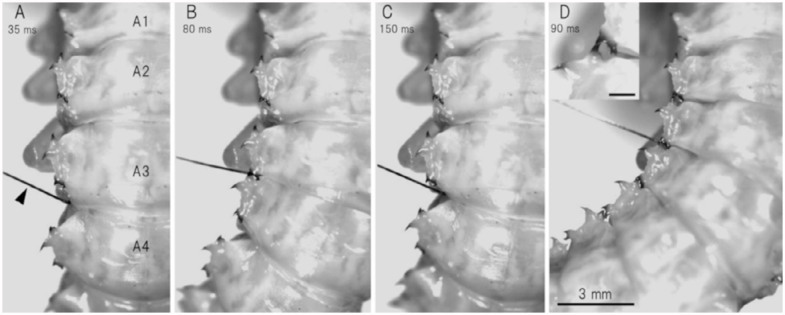
Dorsal views of the closure of single (A–C) and multiple gin traps of a pupa placed dorsal-side up (D). By touching the lateral area of intersegmental membrane between abdominal segment A3 and A4 with a thin wire (arrowhead), jaws start to close at 35 ms (A), close completely at 80 ms (B), and reopen 150 ms after the onset of stimulation (C). (D) Stronger stimulation of the same region evokes the closure of multiple gin traps 90 ms after the onset of stimulation. A1–A4, abdominal segments A1–A4. Inset shows a close-up view of closed jaws (bar = 0.5 mm). High quality figures are available online.

The pupal abdomen consists of nine segments that are numbered A1–A9. There are three claw-shaped processes or spines on each lateral flange of segments A1–A7. The anterior and posterior processes are associated with a row of sclerotized teeth that form a jaw. The posterior and anterior jaws on subsequent segments make a pinching device known as a gin trap (Figure 1). The third middle process without teeth is not involved in the pinching mechanism.

### Simple gin-trap closure response

**Figure 2.**
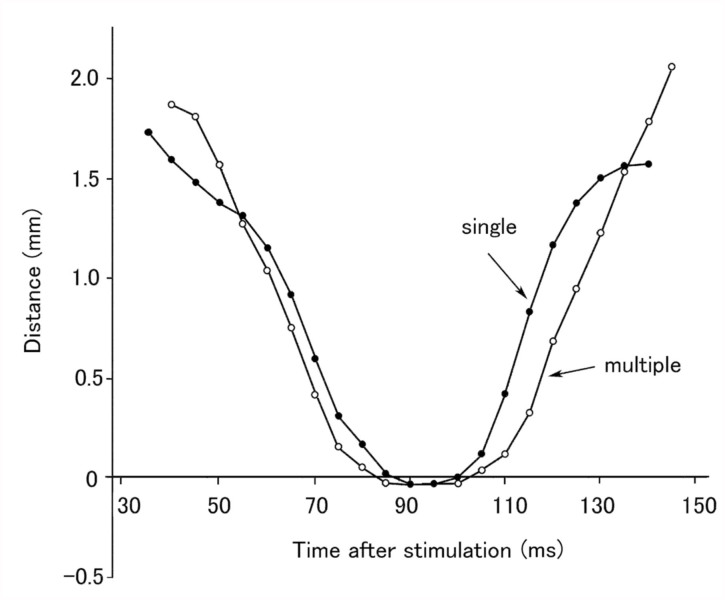
Time courses of two types of gin trap closing-opening responses involving a complete closure of single and multiple gin traps. Distances between tips of the two jaws in segments A2 and A3 are plotted as a measure of the response. Negative values indicate a reversal of the position of the tips due to the occlusion of the jaws. Responses with the same delay time were selected. High quality figures are available online.

**Figure 3.**
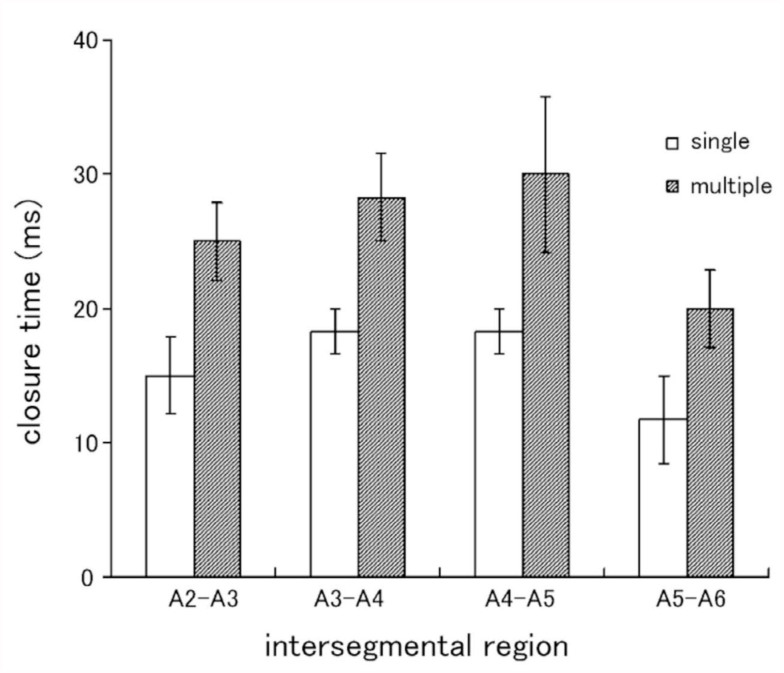
Durations of closed states of gin traps in the two grades of responses. Responses were induced by brushing the intersegmental region between adjacent segments of A2–A6. Means ±SEM obtained from a pupa are shown (n = 10). High quality figures are available online.

A small area in the lateral region of the intersegmental membrane between the posterior margin of an abdominal segment and the spiracle of the next segment was most sensitive to tactile stimulation. A gin-trap closure response could be readily evoked by prodding the area with a thin filament (bending force, 0.6 mN) or weakly brushing the area and surrounding area with a fine brush. Similar tactile stimulation applied to other abdominal regions away from the sensitive area elicited no gin-trap response. A relatively weak stimulation usually elicited the closure of the single gin trap (e.g., Figure 1A–C), while a strong stimulation evoked the closure of multiple gin traps (e.g., Figure 1D). When the intersegmental area between the third and fourth segments was stimulated, the anterior jaw on the fourth segment started to move anteriorly approximately 35 ms after the onset of stimulation (Figure 1A), just occluded with the posterior jaw on the third segment at 80 ms (Figure 1B), then started to move posteriorly at 105 ms, and finally stopped moving 150 ms after the onset of stimulation (Figure 1C). Thus, the rapid closure of the gin trap was followed by a rapid reopening after a brief intermission of approximately 25 ms. The mean latency of the response (the start of the anterior movement) was 33 ± 6 ms (n = 10). When a large gin-trap response to a strong stimulus occurred, the abdomen bended maximally toward the side of stimulation; in addition, two or three traps adjacent to the stimulated trap usually closed completely, while the remaining traps closed partially (Figure 1D). Figure 2 shows the time courses of the gin-trap closing-opening responses in which the distances between the tips of the two jaws are plotted as a measure of the response. The stimulated traps closed earlier and reopened later during the large response involving multiple gin traps than during a small response involving a single trap. The negative value of the distance means a reversal of the position of the tips at the occlusion of jaws (see Figure 1 inset). If the period of the negative value was defined as the duration of a closed state, the duration during a large response was 5 ms longer than the duration during a small response. Figure 3 shows the mean durations of closed states of different gin traps during the two grades of responses. The mean durations of closed states appeared to be maximal in the gin traps lying between A4 and A5 or A3 and A4, which are larger than the other segments. The mean durations during large responses were approximately 10 ms longer than those during small responses. When the two jaws between A3 and A4 successfully pinched an object (adult tibial segment), the closed state was significantly prolonged to 65–150 ms (mean ± SEM, 104.3 ± 12.6 ms, n =10).

**Figure 4.**
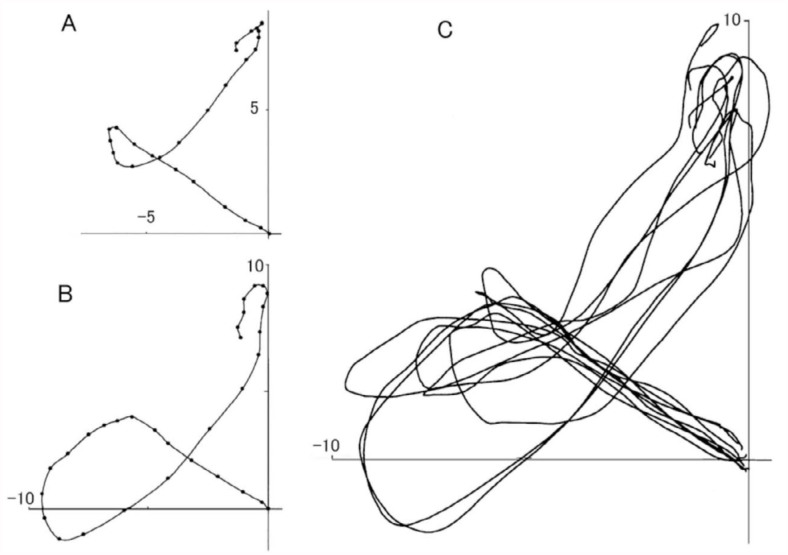
Trajectory patterns of the last abdominal segment during gin-trap closing-opening responses. Positions of the last segment during small (A) and large (B) responses are plotted at 5-ms intervals (dots). (C) = superimposition of eight trajectories of the abdominal motion induced by brushing the intersegmental region between A3 and A4. High quality figures are available online.

To analyze the trajectory patterns of the abdomen during the gin-trap responses, a pupa was usually placed ventral-side up, and the position of the last abdominal segment on a posterior view was plotted every 5 ms. Figure 4 shows typical trajectory patterns of small and large gin-trap closure responses. The abdominal segment moved laterally in an arc during the closing phase and turned back medially and ventrally to reach the upper (ventral) position from the original resting position during the opening phase. Thereafter, the segments slowly returned to the original position in 1 second. The trajectories of the closing and opening phases usually crossed at a midpoint of their length.

### Complex gin-trap closure response

**Figure 5.**
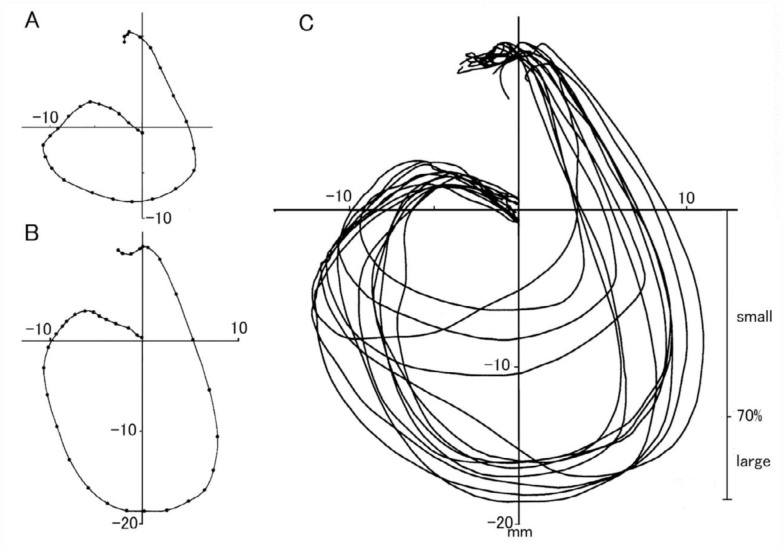
A variety of trajectory patterns of abdominal rotations following gin-trap closure responses to stimulation of the intersegmental area between A3 and A4. Positions of the last abdominal segment during a small (A) and large (B) rotation are plotted at 5-ms intervals (dots). (C) = superimposition of 13 trajectories of abdominal rotations, including the trajectories shown in A and B. Rotations are divided into two classes, small or large, according to whether the amplitude of vertical component of the rotation was lesser or greater than 70% of the maximum amplitude of the vertical component of the largest rotation. High quality figures are available online.

**Figure 6.**
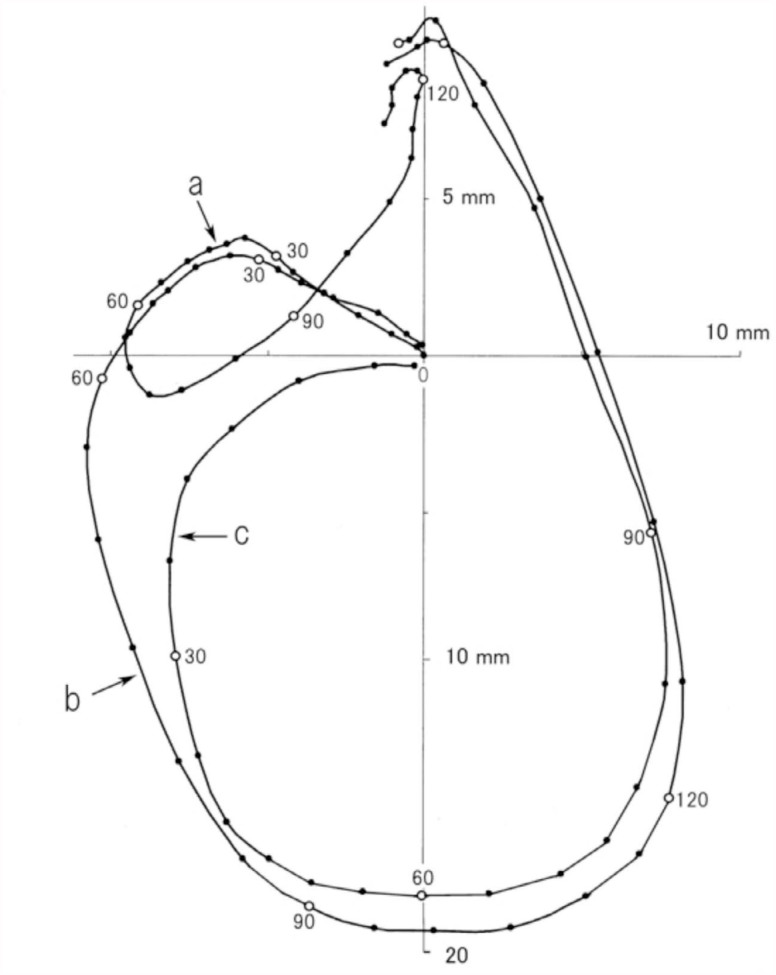
Comparison of typical trajectory patterns of abdominal movements in the simple gin-trap closing-opening response (a) and abdominal rotations that are induced by brushing the intersegmental area (b) and the foreleg (c). Rotation onset is defined as time = 0. High quality figures are available online.

A strong stimulation of the sensitive area of the intersegmental membrane often induced a small or large rotation of the abdomen rather than the simple gin-trap closing-opening response. The trajectory patterns of many rotational responses revealed that the rotational responses were graded and had highly variable horizontal and vertical amplitudes of the rotational movements (Figure 5). The variability of the rotational responses contrasts with the relative consistency of rotational responses induced by the stimulation of an appendage. Upon initial observation, a large rotation of the abdomen that was induced by stimulating the intersegmental region appeared to be similar to the abdominal rotation induced by stimulating an appendage; however, the temporal and spatial patterns of the two rotation types apparently differed (Figure 6). The abdominal rotation induced by stimulating specific abdominal regions appeared to have a relatively slow initial phase of rotation followed by a rapid later phase. The shoulder-shaped trajectory course of abdominal movement during the initial phase was very similar to the arc-shaped trajectory course during the closing phase of the gin-trap response. The last abdominal segment reached only to a point several millimeters away from the starting point at 60 ms after the onset of rotation (Figure 6b), as it did during the phase of the gin-trap response (Figure 6a). In contrast, the stimulation of an appendage (a leg) induced a simple rapid rotation that had no slow initial phase and could reach the halfway point of rotation 60 ms after the onset of rotation (Figure 6c). The duration of the closed state of the gin trap was prolonged to 50–60 ms when a large abdominal rotation occurred in response to stimulation of an intersegmental region (data not shown).

**Figure 7.**
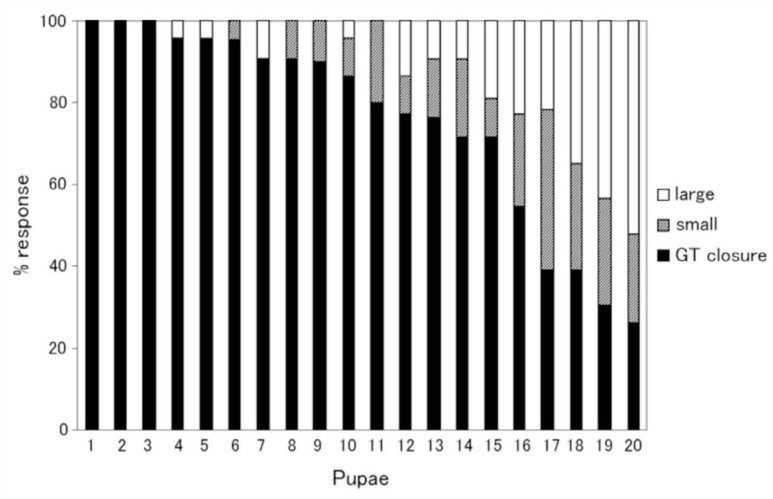
Variability of three types of abdominal movements among different pupae. Abdominal movements induced by stimulation of the intersegmental area are classified into simple gin-trap closing-opening responses (GT closure), small rotations (small), and large rotations (large). Twenty stimuli were delivered to each pupa. High quality figures are available online.

The occurrence of abdominal rotations following the gin-trap closure phase varied from pupa to pupa, even though pupae of the same age (one day old) were used (Figure 7). A rotation was classified as small or large when the amplitude of the vertical component of a rotation was lesser or greater than 70% of the maximal amplitude of the vertical component of the largest rotation in the pupa. A few pupae always exhibited a gin-trap closing-opening response even when a stronger brushing was applied to the sensitive area of intersegmental membrane. Meanwhile, in the most sensitive pupa, half of the responses to the stimulus were classified as large. The remaining pupae were in between the other two groups. The probability of a small response was usually < 25%. The occurrence of abdominal rotations in response to stimulation of gin traps between A3 and A4 or A4 and A5 seemed to be somewhat larger than that in response to stimulation of the gin traps of other segments; however, these differences were not examined systematically.

## Discussion

The particular area of the intersegmental membrane near a gin trap had many campaniform sensilla (Kurauchi et al. 2011), and gentle brushing of this area with a fine brush readily induced a response. Potential predators of the pupa in nature include carnivorous insects, centipedes, and spiders (Hinton 1946). Since the pupae usually have long appendages (i.e., antennae or legs) that are often covered with many sensory and protective hairs, the hairy part of their bodies may be most suitable for inducing the gin-trap closing-opening response of the pupa. In turn, the gin trap may be adapted to pinch the appendages of the potential enemies. Interestingly, the traps usually snapped shut for only a split second (Figures 1 and 2) and did not remain closed for longer than 150 ms even when the jaws successfully bit an appendage. This suggests that the pupa cannot cause serious damage to an attacker. The gintrap closure response may mainly function to startle or deter attackers (Hinton 1946; Eisner and Eisner 1992). If a gin trap remained closed for any length of time while they held an attacker, the attempts of the attacker to free itself could result in serious injury to the pupa (Hinton 1946). The abdominal rotations that often followed the closure of gin traps (Figure 5) may make the pupa turn its dorsum toward the enemy (Ichikawa et al. 2012); the dorsum, which is fringed with many spines, probably functions as a shield. Since the soft intersegmental region is vulnerable to attack by parasitoids (Gross 1993), closing this vulnerable region may also be effective against parasitoids.

The magnitudes of abdominal rotations that occurred after gin-trap closure varied significantly (Figure 5); this graded response contrasts with the stereotypical response induced by stimulating a cephalic or thoracic appendage (Ichikawa et al. 2012). To account for the stereotypical abdominal rotation patterns observed, we propose that the central nervous system (abdominal ganglion) may possess a neuronal mechanism that generates a motor pattern that rotates the abdomen in one (i.e., clockwise or anticlockwise) direction. However, some modification of the single pattern generator model is needed, because this model cannot explain why some pupae exhibited a small but significant difference in the trajectory patterns of their abdominal rotations when different parts of the body (appendages) were stimulated (Ichikawa et al. 2012). *Z. atratus* pupae have nine abdominal segments numbered A1–A9; each abdominal segment from A2–A6 has four longitudinal (intersegmental) muscle bundles that move the abdomen. It is reasonable to suppose that the magnitude of an abdominal rotation may depend on the number of abdominal segments involved in the rotation. In a preliminary experiment, the trajectory patterns of abdominal rotations became significantly small when some caudal segments of the abdomen were immobilized by surgical transection of the ventral nerve cord between A3 and A4 or A4 and A5. Thus, the graded rotational responses observed in the present study may be due to the difference in the number of abdominal segments activated. It seems likely that each abdominal ganglion from A2–A6 has a pattern generator producing a clockwise or anticlockwise rotation and that all or some pattern generators may be activated depending on the origin and strength of mechanosensory signals. For example, a descending sensory signal originating from a cephalic or thoracic segment usually activates all anticlockwise pattern generators to mobilize all abdominal muscles (e.g., Figure 6c), while a weak signal from an abdominal segment may activate a fraction of the pattern generators to recruit muscles in a few segments near the site of stimulation (Figure 5A). This multiple pattern generator model possibly overcomes the weakness of the single pattern generator model, because small innate variability (fluctuation) of motor patterns caused by individual pattern generators may summate to become significant in a multiple pattern generator system.

The activities of central pattern generators are generally modulated by sensory feedback mechanisms (Delcomyn 1980; Marder and Buchner 2001). Because the closure time of the gin traps was significantly prolonged when the jaws trap an object, a feedback control mechanism of pattern generation may exist. Several campaniform sensilla found in the jaws (Kurauchi et al. 2011) may be involved in such a feedback control mechanism of the putative pattern generators. Electrophysiological studies may reveal the location and properties of the pattern generators and their sensory control mechanism.
